# Hypothalamic‐pituitary‐adrenal axis and depression symptom effects of an arginine vasopressin type 1B receptor antagonist in a one‐week randomized Phase 1b trial

**DOI:** 10.1002/brb3.628

**Published:** 2017-02-09

**Authors:** David A. Katz, Charles Locke, Nicholas Greco, Wei Liu, Katherine A. Tracy

**Affiliations:** ^1^ AbbVie Inc.North ChicagoILUSA

**Keywords:** antidepressants, biological markers, clinical trials, depression, measurement/psychometrics, mood disorders, pharmacotherapy

## Abstract

**Background:**

Arginine vasopressin 1B receptor (V_1B_) antagonists may have utility for the treatment of major depressive disorder (MDD).

**Methods:**

The V_1B_ antagonist ABT‐436 (*N *= 31) or matching placebo (*N *= 20) was administered to MDD subjects for 7 days. The main study objectives were to assess the safety and hypothalamic–pituitary–adrenal axis (HPA) effects of ABT‐436 in MDD subjects. MDD symptoms were assessed using the 17‐item Hamilton Depression Rating Scale (HAM‐D‐17) and the subject‐rated Mood and Anxiety Symptom Questionnaire (MASQ).

**Results:**

The most prevalent safety finding associated with ABT‐436 800 mg QD was increased mild‐moderate diarrhea (68% v 5%, *p *< 0.001). Increased nausea (26% v 5%, *p* < 0.10), decreased systolic blood pressure (3.15–3.44 mmHg, *p* < 0.10) and increased heart rate (3.42–4.01 bpm, *p* < 0.05) were also associated with ABT‐436 800 mg QD. Basal HPA activity measured by 24‐hr urine total glucocorticoids was 25% lower with ABT‐436 than placebo (*p* < 0.001). The reduction was, on average, larger in subjects with higher baseline urine total glucocorticoids. Results on plasma adrenocorticotrophic hormone (ACTH), urine, serum and saliva cortisol, and saliva cortisone also showed basal HPA attenuation with ABT‐436. Dynamic HPA activity measured by plasma ACTH and serum cortisol responses to corticotrophin releasing hormone (CRH) were 30–46% lower in ABT‐436 subjects (all *p* < 0.001). Each ABT‐436 subject showed response to CRH in or near the baseline range of responses. ABT‐436 was associated with more favorable symptom changes on two of five MASQ subscales (estimated effect size 1.47–1.86, *p* < 0.01) but not on HAM‐D‐17.

**Conclusions:**

The results support further clinical study of the antidepressant potential of ABT‐436.

## 1 Introduction

Major depressive disorder (MDD) has 16.9% lifetime and 6.8% 12‐month prevalence (National Comorbidity Survey, 2007). MDD treatment costs have increased, with minimal improvements in quality of care (Fullerton, Busch, Normand, McGuire, & Epstein, 2011). First‐line treatment is associated with limited effectiveness: only 37% of patients achieve remission and 16% do not tolerate treatment (Rush et al., 2006). Medication switching and augmentation characterize subsequent MDD treatment, with decreasing effectiveness at each successive treatment step (Rush et al., 2006). Augmentation therapy with atypical antipsychotics has become much more common, but has not provided improved remission (26–36% vs. 35% for previous augmentation strategies) (Rush et al., 2006) and is associated with increased intolerance (McDonagh et al., 2010). MDD patients with comorbid anxiety or substance use disorders are less likely to achieve remission (Trivedi et al., 2006). There remains considerable need for novel MDD drugs with increased effectiveness, particularly for patients who do not achieve remission with first‐line treatment or who have comorbid anxiety or substance use disorders.

Antagonism of the arginine vasopressin (AVP) 1B receptor (V_1B_) has potential to provide clinical benefit for MDD patients. AVP, a peptide hormone released from the hypothalamus, regulates HPA activity via V_1B_ activation at the anterior pituitary. AVP weakly induces adrenocorticotrophin hormone (ACTH) release and strongly potentiates corticotrophin releasing hormone (CRH)‐induced ACTH release (Dinan & Scott, 2005). Chronic dysregulation of the HPA axis is observed in a subset of MDD patients (Dinan & Scott, 2005). AVP is increased in about 25% of MDD patients (van Londen et al., 1997). HPA responsiveness to AVP is increased in MDD while responsiveness to CRH is decreased (O'Keane, Frodl, & Dinan, 2012). Chronic stress up‐regulates AVP and pituitary V_1B_ expression in man, leading to higher cortisol levels (O'Keane et al., 2012). HPA axis normalization, a potential consequence of ABT‐436 treatment, has been observed to predict lower relapse risk in remitted MDD patients (Targum, 1984) and precede clinical response to antidepressants in some patients (Schüle et al., 2009). V_1B_ antagonism in limbic brain regions may also contribute to ABT‐436 efficacy. In animal models of depression and anxiety, both HPA axis and limbic V_1B_ receptors contribute to effects demonstrated by V_1B_ antagonists (Roper, O'Carroll, Young, & Lolait, 2011).

ABT‐436 is a potent (functional K_b_ 0.45 nmol/L for *in vitro* intracellular calcium release) and selective (≥250‐fold vs. other human AVP/oxytocin receptors) V_1B_ antagonist (Wernet et al., 2008). It has demonstrated effects in animal models of antidepressant and anxiolytic activity, including stress‐induced hyperthermia in mice, rat forced swim test, locomotor activity in olfactory bulbectomized rats, and Vogel conflict test in rats (van Gaalen et al., 2008).

Single doses of ABT‐436 up to 1600 mg (Liu, Katz, Tracy, et al., 2015a) and multiple doses up to 1500 mg QD for 14 days (Katz et al., 2016; Liu, Katz, Tracy, et al., 2015b) were administered to healthy adults. A maximum tolerated dose was not established. Transient reduced levels of plasma ACTH and serum cortisol were associated with single (AbbVie, unpublished observations) and multiple doses (Katz et al., 2016) of ABT‐436 as low as 150 mg ABT‐436. Food did not have meaningful effect on the pharmacokinetics of a single dose of 200 mg ABT‐436 (Liu, Katz, Tracy, et al., 2015a). Mild gastrointestinal intolerance was more common with ABT‐436 than with placebo and showed dose dependence. There was evidence for ABT‐436 related increases and decreases (at different times of day) on mean changes of systolic blood pressure and ABT‐436 related pulse rate increases (Katz et al., 2016).

We sought to learn whether ABT‐436 would show similar effects in MDD subjects as observed in healthy adults. Particular attention was paid to whether MDD subjects would maintain adequate cortisol levels despite HPA axis attenuation by ABT‐436. ABT‐436 (*N *= 31) or matching placebo (*N *= 20) was administered to MDD subjects for 7 days. The main study objectives were to assess the safety and HPA effects of ABT‐436 in MDD subjects. Response to a CRH challenge was included during the trial to assess whether ABT‐436 might cause secondary adrenal insufficiency (inability of the HPA axis to respond in a crisis situation). An additional pre‐specified study objective was to learn whether ABT‐436 reduced MDD symptoms, assessed using the 17‐item Hamilton Depression Rating Scale (HAM‐D‐17) and the Mood and Anxiety Symptom Questionnaire (MASQ) (Watson et al., 1995).

## 2 Materials and Methods

This randomized, double‐blind, placebo‐controlled parallel group study was conducted at five sites from May to September 2011, following ICH GCP guidelines and ethical principles of the Declaration of Helsinki. Copernicus Group IRB (Research Triangle Park, NC) approved the protocol and informed consent forms. The trial was registered (http://clinicaltrials.gov/show/NCT01380704).

Adult male and female subjects (*N *= 52) were between 18 and 55 years of age and in general good physical health based on medical history, physical and neurological examinations, vital signs, 12‐lead electrocardiogram (ECG), clinical laboratory tests, and the Columbia Suicide Severity Rating Scale. Subjects had a primary diagnosis of MDD according to the Diagnostic and Statistical Manual of Mental Disorders–Fourth Edition, Text Revision, current mild‐to‐moderate depressive symptoms assessed with the Clinical Global Impression of Severity, and were not taking any antidepressants or other psychotropic medications. Psychiatric diagnoses were confirmed, using the Mini‐International Neuropsychiatric Interview version 6.0.

Subjects had not used any other medication within 4 weeks prior to the start of confinement, unless the dose had been stable for at least 4 weeks, no dose change was anticipated during the study, and the medication was specifically allowed per the protocol. As‐needed use of some medications (e.g., over‐the‐counter analgesics) was allowed per the protocol. Subjects were not current or recent (12 months) nicotine users, or drug or alcohol abusers. Exclusionary psychiatric history included MDD with psychotic features, bipolar disorder, schizophrenia, other psychotic disorder, mental retardation, or a psychiatric disorder secondary to a medical condition. Any Axis II disorder that may have impacted the subject's ability to comply with the protocol was exclusionary. Ongoing psychotherapy was allowed if study participation did not interfere with it.

Subjects were randomized to 7 days dosing (starting Day 1) of 800 mg ABT‐436 QD (*N *= 31) or matching placebo (*N *= 20). The ABT‐436 dose and trial duration were selected based on results of prior clinical studies of ABT‐436 safety, pharmacokinetics and HPA axis effects in healthy adults (Katz et al., 2016; Liu, Katz, Tracy, et al., 2015b). Subjects were screened between 4 and 28 days prior to the first study drug dose. Subjects were confined to the study site from the morning of Day −2 (second day prior to the first study drug dose) until completion of scheduled study procedures on Day 8 (first day after the last study drug dose).

Additional details are provided in Supporting Information Materials and Methods.

## 3 Results

Fifty‐one subjects (33 males, 18 females) completed the confinement portion of the study. Two subjects did not complete the safety follow‐up period. One subject was discontinued from the study prior to receiving any study drug dose, due to an abnormal lab result recognized after randomization. No results from that subject are included herein.

Baseline characteristics are presented in Table S1. Although no statistical comparisons were made, subjects randomized to placebo showed numerical differences compared to those randomized to ABT‐436: longer average current episode duration, higher proportions of prior antidepressant medication use during the current episode and recurrent MDD diagnosis. Prior and concomitant medication use was somewhat more common among ABT‐436 subjects (Table S2). Most concomitant medications among ABT‐436 subjects were added during the study for treatment of gastrointestinal adverse events.

### 3.1 Safety

No deaths, serious adverse events or discontinuations due to adverse events occurred during the study. Treatment‐emergent adverse events (Tables 1 and S3) were more prevalent in the ABT‐436 group (94% v 55%). Diarrhea (68% v 5%), nausea (26% v 5%), and gastrointestinal disorders System Organ Class (77% v 15%) were more prevalent in the ABT‐436 group. Insomnia was more prevalent in the placebo group (0% v 15%). One headache in an ABT‐436 subject was assessed as severe. The investigator assessed all other treatment‐emergent adverse events as mild or moderate in severity. Orthostatic hypotension and tachycardia considered clinically significant were each observed in one ABT‐436 subject and reported as adverse events (Table S3). No other clinical laboratory, vital signs or ECG individual results were considered clinically significant by the investigator. No subject reported suicidal ideation after starting study drug.

**Table 1:** Treatment‐emergent adverse events observed in two or more subjects

	Placebo (%)	ABT‐436 (%)
N	20	31
Any event	11 (55)	29 (94)**
Diarrhea	1 (5)	21 (68)***
Nausea	1 (5)	8 (26)+
Headache	2 (10)	6 (19)
Abdominal pain	0	4 (13)
Flushing	2 (10)	2 (7)
Anxiety	1 (5)	2 (7)
Insomnia	3 (15)	0+
Abdominal discomfort	0	2 (7)
Constipation	0	2 (7)
Dizziness	1 (5)	1 (3)
Dyspepsia	1 (5)	1 (3)

+ *p* < 0.10 vs. placebo; **p < 0.01 vs. placebo; ***p < 0.001 vs. placebo.

The investigator assessed diarrhea with ABT‐436 as mild in severity in 15 subjects, and moderate in severity in 6 subjects. It first occurred on Day 1 or 2 in 16 subjects, and later than Day 2 in 5 other subjects. Diarrhea continued after the first day of occurrence on all or most of days of dosing in 12 subjects, subsided during continued dosing in 6 other subjects, and was reported only on the last day of dosing in 3 other subjects. It reversed within 1 day of dosing cessation in all subjects who reported diarrhea on the last dosing day except one, in whom it reversed 2 days after dose cessation. Diarrhea was accompanied by a related gastrointestinal adverse event, most commonly nausea, in 9 subjects.

Prothrombin time was 0.24 sec shorter (*p* = 0.050), aspartate transaminase was 2.40 U/L higher (*p* = 0.028), and glucose was 0.28 mmol/L lower (*p* = 0.031) with ABT‐436 at Day 8. These mean changes were not considered clinically significant and there were no potentially clinically significant individual outlier values for any of these parameters. No other statistically significant differences on laboratory parameters were observed.

There were trends for decreased systolic blood pressure with ABT‐436, averaged across all times of measurement, during each of Day 1 (−3.44 mm Hg, *p* = 0.069) and Day 6 (−3.15 mm Hg, *p* = 0.052). The largest mean difference from placebo was at Hour 6: −6.14 mmHg on Day 1 and −5.87 mmHg on Day 6. ABT‐436 was associated with larger decreases of systolic blood pressure from supine to standing position at the single time of measurement (*p* = 0.041). The mean orthostatic decrease compared to placebo was 6.31 mmHg larger on Day 1, and 3.55 mmHg larger on Day 7. One ABT‐436 subject experienced an adverse event of orthostatic hypotension, without symptoms such as dizziness. No trends on diastolic blood pressure or pulse rate were apparent.

ABT‐436 was associated with increased heart rate at Hour 4 averaged across Days 1, 4 and 6 (*p* = 0.049). The mean difference from placebo was larger on Days 4 and 6 (3.42–4.01 bpm) than on Day 1 (1.15 bpm). There was a trend for increased heart rate with ABT‐436 at Hour 8 on Day 6 (3.59 bpm, *p* = 0.080). One ABT‐436 subject experienced an adverse event of tachycardia. There were significant treatment‐time interactions (*p* < 0.050) on the P‐R interval, reflecting differences between ABT‐436 and placebo that differed in direction at different times or days. None of the mean changes at single time points was statistically significant or assessed as clinically significant. No other statistically significant differences on ECG parameters were observed.

### 3.2 Pharmacokinetics

ABT‐436 exposure following 800 mg QD doses in subjects with MDD was similar to exposures in healthy subjects under the same dose regimen (Liu, Katz, Tracy, et al., 2015a). The maximum post‐dose plasma concentration on Day 6 was 1190 ± 423 ng/mL (mean ± SD), achieved at 2.1 ± 0.4 hr after dosing. The area under the plasma concentration‐time curve for the 24‐hr dosing interval was 6260 ± 2130 ng*hr/mL. The pre‐dose plasma concentration on Day 7 was 86.9 ± 105 ng/mL.

### 3.3 Basal HPA parameters

Urine total glucocorticoids and cortisol were each normalized by creatinine from the same urine collection for statistical analysis. In 24‐hr urine collections, the cortisol/creatinine and total glucocorticoids/creatinine ratio central values (anti‐logarithms of mean logarithm model‐based estimates from analysis of covariance) were approximately 40% and 25% lower in ABT‐436 subjects. The central values (mcg/mg) for 24‐hr urine cortisol/creatinine ratio were 0.080 in ABT‐436 subjects and 0.134 in placebo subjects (*p* < 0.001). The central values for 24‐hr urine total glucocorticoids/creatinine ration were 2.76 in ABT‐436 subjects and 3.67 in placebo subjects (*p* < 0.001).

**Figure 1:**
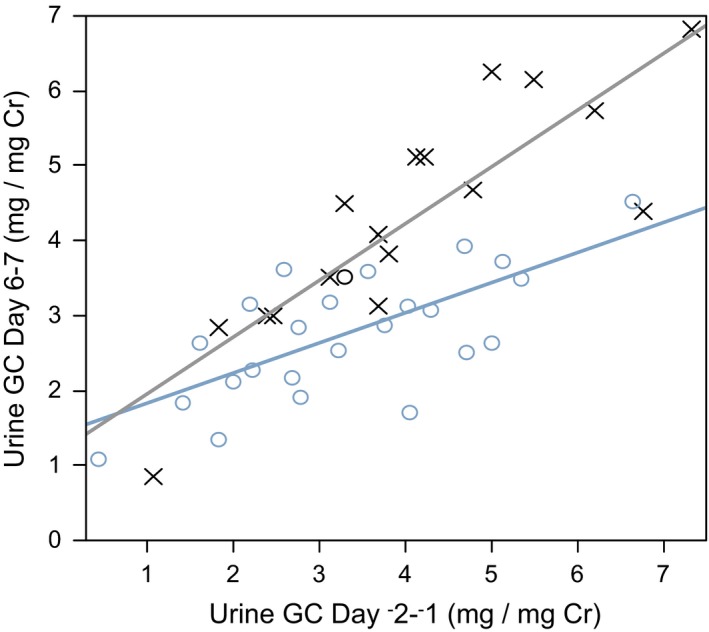
Baseline 24‐hr creatinine (Cr)‐normalized urine total glucocorticoids (GC) as a covariate for the pharmacologic effect of ABT‐436. Placebo: × and grey regression line. ABT‐436: ° and blue regression line. Baseline‐treatment interaction *p* = 0.004

Daily cortisol production, measured by 24‐hr urine total glucocorticoids, during placebo administration showed strong correlation with the same measurement at baseline with a regression slope close to unity (Figure 1). That is, among placebo subjects, cortisol production measured on two separate days one week apart was relatively stable in an individual, as expected. The regression slope for ABT‐436 subjects was smaller (*p* = 0.004). Among subjects with higher baseline daily cortisol production, the observed difference at Day 6 between placebo and ABT‐436 subjects was large. In contrast, little or no difference was observed among subjects with lower baseline daily cortisol production (Figure 1).

**Figure 2:**
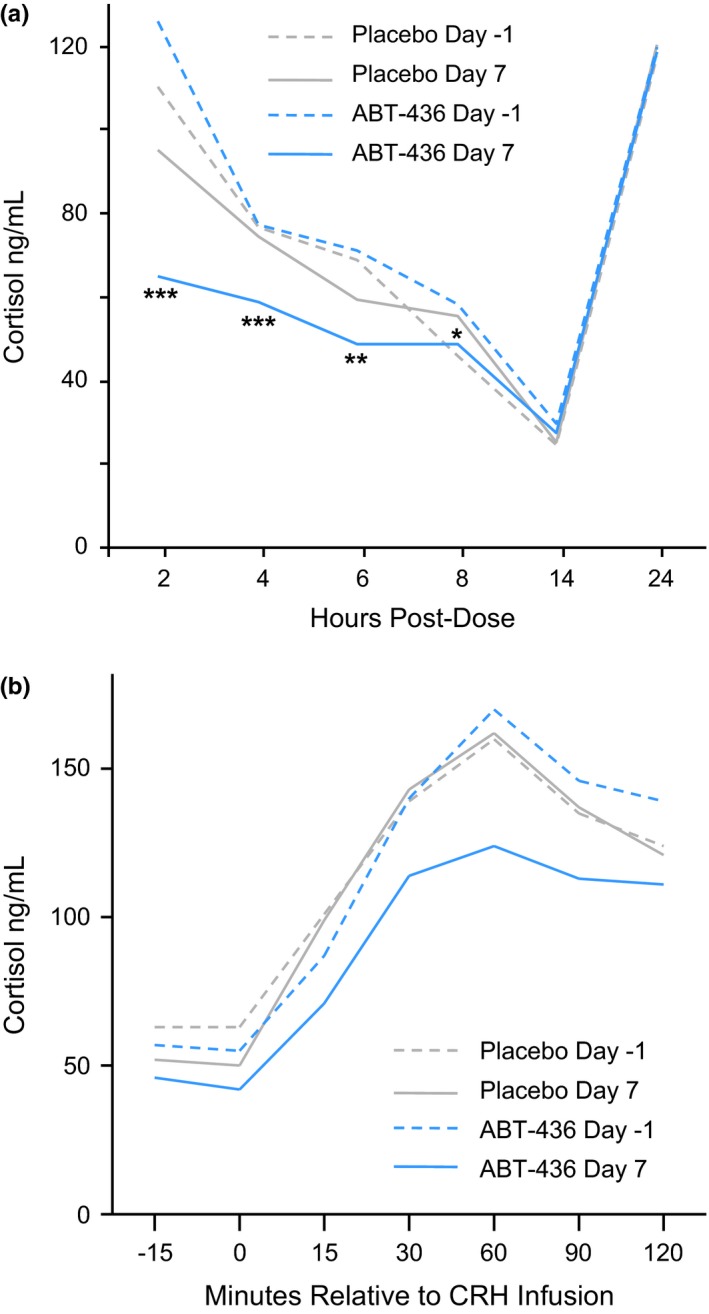
Serum cortisol levels by regimen, day and hours after dosing (Day 6/7) or time‐matched to dosing (Day −2/−1). (a) Basal serum cortisol levels. (b) Dynamic serum cortisol levels following a corticotrophin releasing hormone challenge. *p < 0.05 **p < 0.01 ***p < 0.001

The average (across multiple measurement times during a day) plasma ACTH, serum cortisol and free cortisol levels were approximately 21%, 22% and 37% lower in ABT‐436 subjects compared to placebo subjects (Table 2). Lower plasma ACTH, serum cortisol and free cortisol levels in ABT‐436 subjects were mainly observed up to 8 hr after study drug administration (Figure 2a). The largest differences were at Hour 2: approximately 43% for ACTH, 41% for total cortisol, and 58% for free cortisol. Average adrenal sensitivity values differed by <1% between ABT‐436 and placebo subjects (Table 2). No statistically significant differences were observed at individual times of measurement.

**Table 2:** Central values for blood measures of hypothalamic–pituitary–adrenal axis activity by regimen

	Placebo	ABT‐436	*p* value
N	20	31	
Basal Values			
ACTH (pg/mL)			
Average	16.88	13.32	<0.001
Hour 2	18.39	10.46	<0.001
Hour 4	18.39	15.16	0.012
Hour 6	17.65	12.71	<0.001
Hour 8	17.52	14.69	0.067
Hour 14	9.92	8.35	0.276
Hour 24	22.32	22.61	0.916
Total cortisol (ng/mL)			
Average	62.35	48.68	<0.001
Hour 2	93.98	55.87	<0.001
Hour 4	72.82	51.72	<0.001
Hour 6	57.93	44.12	0.002
Hour 8	53.74	42.79	0.031
Hour 14	23.48	22.63	0.794
Hour 24	117.44	107.84	0.332
Free cortisol (ng/mL)			
Average	6.59	4.17	<0.001
Hour 2	12.50	5.21	<0.001
Hour 4	8.73	4.30	<0.001
Hour 6	6.18	2.88	<0.001
Hour 8	5.22	2.92	0.006
Hour 14	1.39	1.87	0.116
Hour 24	16.60	14.86	0.378
Adrenal sensitivity (ng/pg)			
Average	3.70	3.67	0.902
Hour 2	5.08	5.34	0.634
Hour 4	3.97	3.47	0.170
Hour 6	3.29	3.47	0.639
Hour 8	3.08	2.87	0.487
Hour 14	2.38	2.76	0.367
Hour 24	5.28	4.79	0.246
Dynamic Values (CRH Challenge)			
Maximum ACTH (pg/mL)	42.4	22.8	<0.001
AUC ACTH (pg*hr/mL)	45.1	27.8	<0.001
Maximum cortisol (ng/mL)	123	85.7	<0.001
AUC cortisol (ng*hr/mL)	165	115	<0.001
Adrenal sensitivity (ng/pg)			
Average	3.29	3.76	0.065
+15 min	2.73	3.44	0.012
+30 min	2.99	4.22	<0.001
+60 min	3.37	4.05	0.014
+90 min	3.87	3.70	0.562
+120 min	3.63	3.45	0.537

Adrenal sensitivity is ng cortisol / pg ACTH.

ACTH, adrenocorticotropin hormone; CRH, corticotrophin releasing hormone.

Awakening saliva cortisol and cortisone central values were approximately 40% and 30% lower in ABT‐436 subjects compared to placebo subjects (Table S4). The average of three morning samples (awakening and 0.5 and 1 hr after awakening) saliva cortisol and cortisone central values were approximately 27% and 19% lower in ABT‐436 subjects compared to placebo subjects. The average saliva cortisol and cortisone awakening responses were approximately 20% and 19% lower in ABT‐436 subjects compared to placebo subjects. Afternoon saliva cortisone central value was approximately 25% lower in ABT‐436 subjects compared to placebo subjects. A similar difference was not observed for saliva cortisol. The average saliva cortisol and cortisone diurnal amplitudes were approximately 23% and 16% lower in ABT‐436 subjects compared to placebo subjects.

No statistically significant differences between ABT‐436 and placebo subjects were observed on AVP, copeptin, DHEA‐S, androstenedione, testosterone or estradiol.

### 3.4 Dynamic HPA parameters

Mean ACTH and cortisol responses to CRH infusion were attenuated with ABT‐436 (Table 2). Maximum change and AUC were each reduced approximately 30% for cortisol (Figure 2b). The mean reductions for ACTH were somewhat larger: approximately 46% on maximum change and 38% on AUC. Dynamic adrenal sensitivity following CRH infusion was on average approximately 14% higher in ABT‐436 subjects compared to placebo subjects (Table 2). Dynamic adrenal sensitivity was increased in ABT‐436 subjects during the acute rise of ACTH and cortisol in the 60 minutes following CRH infusion.

Individual cortisol maximum changes and AUCs following CRH infusion were generally within the ranges observed during time‐matched CRH infusion at baseline (normative ranges). Eleven of 31 ABT‐436 subjects, compared to 0 of 20 placebo subjects, showed individual peak cortisol values during CRH infusion below the normative range. Ten of the 11 subjects who showed low peak cortisol levels showed cortisol maximum changes and AUCs during CRH infusion within the normative ranges. One subject showed a peak cortisol level of 110 ng/mL (5% below the normative range) and also showed maximum change and AUC during CRH infusion 9–24% below the normative ranges.

### 3.5 Depression symptoms

At baseline, the mean MASQ subscale scores (scale 1–5) followed the pattern expected for a study in which subjects were recruited based on a MDD diagnosis and not for anxiety diagnosis or symptoms. Subjects had an average baseline score 3.7 on Anhedonic Depression, comprised of symptoms exclusive to MDD. Average baseline General Distress subscale scores decreased with decreasing weighting on MDD: Depressive Symptoms (2.9) > Mixed Symptoms (2.5) > Anxiety Symptoms (1.8). The lowest average baseline score 1.2 was on Anxious Arousal, comprised of symptoms exclusive to anxiety disorders.

**Figure 3:**
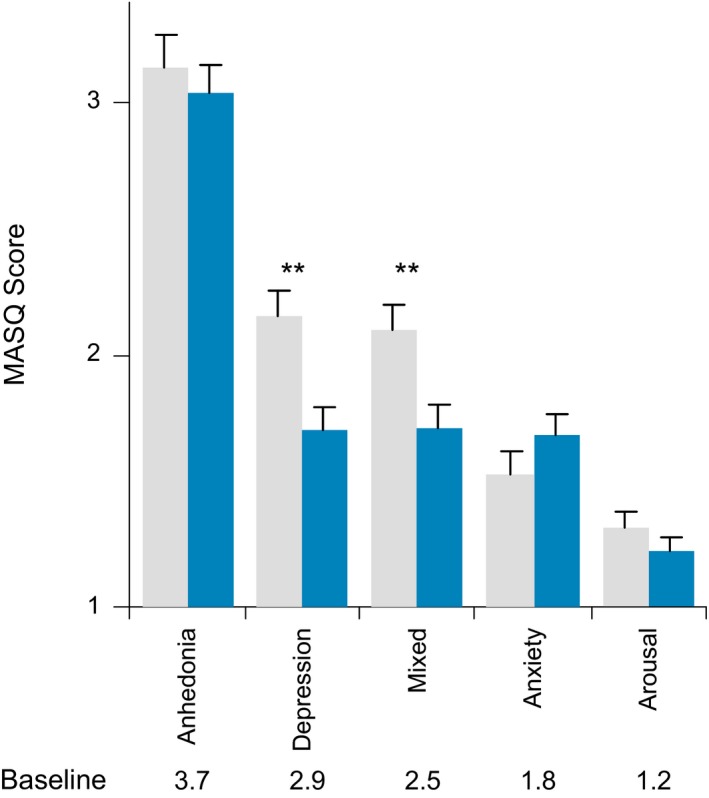
Mood and Anxiety Symptom Questionnaire (MASQ) subscale final scores by regimen. **p < 0.01

ABT‐436 subjects showed favorable symptom responses, compared to placebo subjects, on the MASQ General Distress–Depressive Symptoms (estimated effect size 1.86, *p* = 0.002) and General Distress–Mixed Symptoms (estimated effect size 1.47, *p* = 0.006) subscales (Figure 3). No differences between groups were observed on the other MASQ subscales.

The mean baseline HAM‐D‐17 score was 17.6. The least squares mean final scores were 12.6 for ABT‐436 and 13.2 for placebo (P NS). Analyses on the Diurnal Variation item revealed no clear difference between regimens.

## 4 Discussion

The safety profile of ABT‐436 800 mg QD in MDD subjects was similar to that observed in healthy adults (Katz et al., 2016; Liu, Katz, Tracy, et al., 2015b). The most prevalent safety findings associated with ABT‐436 800 mg QD were increased mild‐moderate diarrhea (68% vs. 5%) and increased nausea (26% vs. 5%). Decreased systolic blood pressure (3.15–3.44 mmHg) and increased heart rate (3.42–4.01 bpm) were also associated with ABT‐436 800 mg QD. A dose‐dependent increase of diarrhea and other gastrointestinal adverse events was previously observed in healthy adults. There was some evidence for tachyphylaxis of blood pressure and heart rate effects with longer dosing in healthy adults (Katz et al., 2016). Additional safety findings could appear with longer dosing in larger studies.

Results of this study replicate and extend the observation of basal HPA (in absence of a particular stressor) attenuation by ABT‐436 in healthy adults (Katz et al., 2016). There were similar reductions of urine total glucocorticoids and cortisol, plasma ACTH and serum cortisol in MDD subjects as in healthy adults to whom 800 mg ABT‐436 QD was administered. There was no compensatory increase of AVP in either MDD subjects or healthy adults. Basal HPA attenuation has now also been observed as decreased serum free cortisol and saliva cortisol and cortisone, parameters that were not rigorously measured in prior studies.

Measured by 24‐hr urine total glucocorticoids, the magnitude of basal HPA attenuation associated with ABT‐436 was relatively large among MDD subjects with higher (but within normal range) pre‐dosing HPA activity. In contrast, little or no basal HPA attenuation was associated with ABT‐436 among MDD subjects with lower (also within normal range) pre‐dosing HPA activity. This relationship between pre‐dosing HPA activity and basal HPA attenuation was also observed in *post hoc* analysis of healthy adult results (AbbVie, data on file). This relationship suggests that basal HPA attenuation by ABT‐436 in a patient with endogenously low HPA activity is not likely to exacerbate risk for adrenal insufficiency.

ABT‐436 has now also demonstrated dynamic HPA attenuation (in response to CRH infusion). Both ACTH and cortisol responses to CRH infusion were reduced. Adrenal sensitivity (level of cortisol divided by level of ACTH) was higher in ABT‐436 subjects compared to placebo subjects during the acute cortisol increase following CRH infusion. Each individual response to CRH infusion in ABT‐436 subjects was within, or close to, the normative range for such responses in MDD subjects based on the pre‐dosing results of this study. Both increased adrenal sensitivity following CRH infusion and observed individual cortisol responses to CRH infusion suggest low risk of inadequate dynamic HPA activity associated with ABT‐436.

While safety and pharmacodynamics were the main objectives of this study, symptom changes were measured on HAM‐D‐17 and MASQ (Watson et al., 1995). A cross‐sectional analysis of symptom severity measured using MASQ showed correlation with cortisol levels (Veen et al., 2011). Since ABT‐436 lowers cortisol levels, prior to this study we hypothesized that MASQ could be sensitive to detect symptom changes, if any, associated with ABT‐436. Observations of more favorable symptom changes with ABT‐436, compared to placebo, on two of five MASQ subscales are consistent with the hypothesis that MASQ could be sensitive to ABT‐436. Favorable symptom changes with ABT‐436, compared to placebo, were not observed on the HAM‐D‐17, on which a difference of symptom change at one week is not typically observed for most antidepressants.

Interpreting these observations in relation to ABT‐436's antidepressant efficacy potential is limited by multiple factors. Diarrhea and nausea could have functionally unblinded subjects, leading them to endorse lower scores on the MASQ. We consider that unlikely, as there were no numerical differences on the MASQ subscale scores between ABT‐436 subjects who did and did not experience diarrhea or nausea. The study subjects had on average milder baseline MDD symptoms than typical MDD trial subjects or MDD patients who could be expected to seek treatment. Subjects were confined throughout the study, which could confound antidepressant efficacy assessment. Study drug was administered for only one week. MASQ was not designed for symptom change measurement (Watson et al., 1995), and there are only sparse data showing score changes during antidepressant treatment (Dichter et al., 2005; Tomarken et al., 2004). Baseline differences between regimens on current episode duration, prior antidepressant use or recurrent MDD diagnosis could have confounded the analysis. This small study was powered to detect differences of basal HPA activity, not symptom changes. The statistically significant investigator effect in the analysis might indicate differences of population or administration between sites. Despite these limitations, the results of pre‐specified analyses showed statistical significance (even applying a strict Bonferroni correction for the six specified analyses) for more favorable symptom changes with ABT‐436, supporting a potential for antidepressant efficacy.

## 5 Conclusions

ABT‐436 has demonstrated robust pharmacologic attenuation of basal HPA activity in both MDD subjects and healthy adults, and also dynamic HPA attenuation in MDD subjects. ABT‐436 seemed to be associated with favorable depressive symptom changes at one week in MDD subjects. Its main limitation appears to be incidence of mild‐moderate diarrhea. Based on the present results, a traditional MDD clinical trial in subjects with more severe depressive symptoms is warranted to further explore the potential benefit of ABT‐436 for treatment of MDD.

## Conflict of Interest

All authors are (or were at the time of the work) employees of AbbVie Inc and may hold AbbVie stock or stock options. Katz is a corporate officer and a major stockholder of Sparrow Pharmaceuticals, outside the submitted work, Katz is an inventor on pending patent PCT/EP2013/055147 that is licensed to AbbVie Inc.
