# Supplementary material for: Hypothalamic‐pituitary‐adrenal axis and depression symptom effects of an arginine vasopressin type 1B receptor antagonist in a one‐week randomized Phase 1b trial
Source: Brain Behav. 2017 Feb 9;7(3):e00628. doi: 10.1002/brb3.628 (PMC5346517; doi:10.1002/brb3.628)
Supplement: Supplementary file 1 [file BRB3-7-e00628-s001.docx]

**SUPPLEMENTARY MATERIALS**

**Subject Eligibility and Randomization**

No minimum HAM-D-17 scale was required, to avoid confounding of HAM-D-17 score change by inflation of the baseline score to meet an inclusion criterion. Subjects were randomized from a single schedule across all sites. The randomization was unbalanced (three ABT-436 to two placebo) so that there would be a larger number of ABT-436 subjects for exploration of relationships between baseline HPA axis biomarkers and subsequent ABT-436 effects. An unblinded pharmacist dispensed study drug after receiving a randomization number from AbbVie. All other site personnel did not have access to the randomization sequence and were blinded to treatment assignment throughout the study.

**Procedures (Supplemental Figure S1)**

During confinement, each site determined a lights-on time, which was the time that subjects were awakened each day if they did not wake earlier. Dosing was at one hour after lights-on time for all subjects.

Safety was assessed throughout the study. Safety procedures included physical and neurological examinations, vital signs, ECGs, clinical laboratory tests, pregnancy tests, adverse event monitoring, and the C-SSRS. There were outpatient safety follow-up visits on Days 14 and 30.

Plasma samples for ABT-436 assay were collected at the following times: just prior to dosing, at 2, 4, 6, 8, 14 hours after dosing on Day 6; just prior to dosing on Day 7.

Basal HPA parameters (i.e., those that measure activity in absence of a particular stressor) were sampled at the following times, and at the corresponding times on Days -2 and -1 as baseline.

- Plasma ACTH, serum cortisol, cortisol binding globulin (CBG): 2, 4, 6, 8, 14 hours after dosing on Day 6; just prior to dosing on Day 7.
- Plasma AVP, copeptin: 6, 14 hours after dosing on Day 6; just prior to dosing on Day 7.
- Serum testosterone, estradiol, dihydroepiandrosterone sulfate (DHEA-S), androstenedione: 14 hours after dosing on Day 6; just prior to dosing on Day 7.
- Urine cortisol, cortisone, tetrahydrocortisol, allotetrahydrocortisol, tetrahydrocortisone, creatinine: 24 hours starting 4.75 hours after dosing on Day 6.
- Saliva cortisol, cortisone: 6, 14 hours after dosing on Day 6; at lights-on, +30 and +60 minutes on Day 7.

During venipuncture, the ACTH sample was collected first, into pre-chilled tubes. AVP and copeptin samples were also collected into pre-chilled tubes. The tubes were immersed immediately in an ice bath. Plasma was separated in a refrigerated centrifuge within 30 minutes, and stored at -70° or below until assay. Blood for cortisol, CBG, testosterone, estradiol, DHEA-S, and androstenedione was allowed to clot for 30-60 minutes at room temperature. Serum was separated, and stored at -70° or below until assay. Urine was kept refrigerated during the collection intervals. At the end of the intervals, the urine was thoroughly mixed and the volume was measured. Isopropanol (10% volume/volume) was added to urine aliquots for steroid measurement. Urine aliquots for steroid and creatinine measurement were stored at -70° or below until assay. Saliva was frozen in Salivette containers (Sarstedt, Newton NC) and stored at -70° or below until assay.

Dynamic HPA parameters (i.e. those that measure response to a stressor) were sampled following CRH administration. An intravenous catheter was placed at 5 hours after dosing on Day 7 and at the corresponding time on Day -1 (baseline). Subjects remained supine from 15 minutes prior to catheter placement until completion of the procedure, except to use the restroom. Subjects were not allowed to sleep, eat or drink (except water) during the procedure. Emotional stimuli during the procedure were limited. Normal saline fluid at approximately 250 mL/hr was infused throughout the procedure. CRH infusion (1 mcg/kg body weight or 100 mcg for subjects with body mass >100 kg; Acthrel, Ferring Pharmaceuticals, Suffern NY) was at 7 hours after dosing. Saline flow was adjusted to maximum from approximately 1 minute before, until approximately 3-4 minutes after, the CRH infusion. The infusion was administered from behind a curtain so the subject did not know exactly when it occurred.

- Plasma ACTH, serum cortisol: -15, 0, +15, +30, +60, +90, +120 minutes relative to CRH infusion.
- Serum tumor necrosis factor alpha, interleukin 6: -15, +30, +60 minutes relative to CRH infusion.

ACTH and cortisol samples were processed as described above. Cytokine samples were processed identically to cortisol samples.

The HAM-D-17 was administered before lunch on Day –2 (baseline) and Day 7 by a trained clinician using a structured interview guide. An additional item on Diurnal Variation was also rated, but not included in the total score.

The MASQ is a 90-item subject-reported measure assessing symptoms that commonly occur in the mood and anxiety disorders (1). Each item is rated from 1 ("not at all") to 5 ("extremely"). The MASQ is comprised of 5 subscales. The Anhedonic Depression subscale (Anhedonia) consists of items assessing symptoms relatively specific to depression. The General Distress: Depressive Symptoms subscale (Depression) contains items that are indicators of depressed mood but do not provide strong differentiation from anxious mood. The General Distress: Mixed Symptoms subscale (Mixed) incorporates items that are indicators of mixed symptoms. The General Distress: Anxious Symptoms subscale (Anxiety) includes items that are indicators of anxious mood but did not provide strong differentiation from depressed mood. The Anxious Arousal subscale (Arousal) is comprised of items assessing anxiety-specific symptoms. Subjects completed the MASQ before lunch on Day –2 and Study Day 7.

**Bioanalysis**

Plasma concentrations of ABT-436 were determined using a validated assay (2).

Plasma ACTH, serum cortisol, CBG, dihydroepiandrosterone sulfate and androstenedione, and urine steroids were measured as previously described (3). Saliva cortisol and cortisone were measured using the previously described serum cortisol assay (3). Serum testosterone and estradiol concentrations were determined using a validated assay (AbbVie, data on file). The following were measured at Quest Diagnostics Clinical Trials (San Juan Capistrano CA): plasma AVP using a direct radioimmunoassay kit (Alpco Diagnostics, Salem NH), plasma copeptin using a sandwich immunoassay (Kryptor Copeptin Ultra-Sensitive, ThermoFisher, Berlin Germany). Urine creatinine was measured at sites’ local clinical laboratories. Tumor necrosis factor alpha and interleukin 6 were measured at Abbott Diagnostics (Abbott Park IL) using a Luminex human cytokine multiplex panel (Life Technologies, Grand Island NY).

**Statistical Analysis**

Values for the pharmacokinetic parameters of ABT-436 were estimated using noncompartmental methods. The maximum observed plasma concentration (C_max_), time to Cmax (T_max_) and concentration at the end of the dosing interval (C_trough_) were determined directly from the plasma concentration-time data. The area under the plasma concentration-time curve for the 24-hour dosing interval (AUC_24_) was calculated by the linear trapezoidal rule.

Adverse events were coded using the Medical Dictionary for Regulatory Activities (MedDRA). The number and percentage of subjects reporting treatment-emergent adverse events were tabulated according to MedDRA preferred term and system organ class with a breakdown by treatment. Tabulations were also provided in which the number of subjects reporting an adverse event (MedDRA term) was additionally broken down by rating (mild, moderate or severe) and by degree of relationship to study drug. Laboratory test values, measurements on vital signs and measurements from ECGs that were potentially clinically significant, according to predefined criteria, were identified.

For clinical laboratory variables, ABT-436 and placebo were compared in a one-way analysis of covariance (ANCOVA) performed on the data for each of Days 2, 5 and 8, with the primary focus on the analysis of Day 8. The last measurement prior to the first dose of study drug was the baseline covariate value.

For blood pressure, pulse rate and quantitative ECG variables, a linear mixed effects model analysis was performed on the scheduled post dose measurements of Day 6. The mixed effects model had effects for baseline value and classification by treatment and time of measurement. The model also had an effect for the interaction of treatment and time of measurement. Such an analysis was also performed for the scheduled measurements of blood pressure and pulse rate during the first 24 hours of the regimen. The baseline value was the average of the scheduled measurements on Days –2 and –1. Within the framework of the model, the estimate and corresponding test on the difference in treatment means was provided for each time of measurement. If the test statistic on interaction was significant at level 0.10, the comparison of ABT-436 and placebo was based upon the tests provided for each individual time. Otherwise, the test of the hypothesis of no difference between the treatments was the test on the treatment main effects. An analysis was performed on the data from the ECGs at 4 hours post dose on Days 1, 4 and 6. This analysis was like that for the ECG variables on Day 6 except that time post dose was replaced by day. For orthostatic changes in blood pressure and pulse rate, an analysis was performed for each of Days 1 and 7. A one way analysis of covariance was employed with the baseline (Day -1) value as the covariate. For each variable, the baseline data of the two groups were described and a two-sample t-test performed.

A repeated measures analysis was performed for each of ACTH, cortisol, free cortisol, AVP, copeptin and four sex steroids. Except for copeptin, the logarithmic transformation was employed in order to reduce the degree of non-symmetry in the probability distributions. In addition, an analysis of covariance was performed for diurnal amplitudes (difference between maximum and minimum observed concentrations) of ACTH, cortisol and free cortisol. For the repeated measures analysis on ACTH, cortisol, free cortisol, AVP, copeptin, DHEA-S and androstenedione, observations were classified by treatment and by time of measurement. The model also had effects for the baseline value and for the interaction of treatment and time of measurement. For a repeated measures analysis, an appropriate structure was selected for the variance/covariance matrix. Within the framework of the selected model, the estimate and corresponding test on the difference in treatment means were provided for each time of measurement. If the test statistic on interaction was significant at level 0.10, the comparison of ABT-436 and placebo was based upon the tests provided for each individual time. Otherwise, the test of the hypothesis of no difference between the treatments was the test on the treatment main effects. For testosterone and estradiol, separate analyses, as described above, were performed for males and females. For each variable a repeated measures analysis was performed on the baseline data to compare the two treatment groups. For testosterone and estradiol, this analysis was done separately for males and females. For the diurnal amplitudes of ACTH, cortisol and free cortisol, a one-way analysis of covariance (ANCOVA) was performed with the baseline value as the covariate. In conjunction, a two-sample t-test was performed on the baseline values.

An analysis was performed for the ratio of the sum of glucocorticoids to creatinine amount and the ratio of cortisol amount to creatinine amount. The logarithmic transformation was employed in order to reduce the degree of non-symmetry in the probability distribution. A baseline value for each variable was determined from the 24-hour urine collection that began on Day –2. For each variable, a one-way ANCOVA was performed with classification by treatment and with the baseline value as the covariate. In conjunction, a two-sample t-test was performed on the baseline values. Analyses like those performed for serum cortisol were provided for saliva concentrations.

Following CRH administration, AUC for the changes of ACTH and cortisol from the pre-infusion level for the two hours of measurement was calculated by the trapezoidal rule. For AUC and the maximum change from the pre-infusion level, one-way ANCOVA like that for the urine variables was performed. However, no transformation of the data was employed for these variables. The baseline covariate was the value of the variable from the CRH infusion on Day –1. A two sample t-test was performed to compare the groups at baseline.

Planned analyses were not performed for tumor necrosis factor alpha and interleukin 6 because they had only a small percentage of values above the limit of quantitation.

A one-way ANCOVA like that for the urine variables was performed for the HAM-D-17 and for the subscales of the MASQ. Although not planned, a factor for investigator was added to the model when substantial variation between sites was recognized. For the HAM-D-17 item on diurnal variation, a three-way frequency table was provided for sub-item A with classification by baseline category, treatment and category on Day 7. Likewise, a three-way frequency table was provided for sub-item B with classification by baseline rating, treatment and Day 7 rating.

The selected sample size provided >90% power for the observed difference in mean between ABT-436 800 mg QD and placebo for urine total glucocorticoids in a prior study, with the variance assumed to be twice that observed in that study (4). Power calculations assumed a 10% dropout rate. The 3:2 randomization ratio was selected, rather than 1:1, to better enable post-hoc exploration for factors that might aid prediction of ABT-436 response.

1. Watson D, Weber K, Assenheimer JS, Clark LA, Strauss ME, McCormick RA (1995): Testing a tripartite model: I. Assessing the convergent and discriminant validity of anxiety and depression symptom scales. *J Abnorm Psych* **104**:3-14.
2. Katz DA, Locke C, Liu W, Zhang J, Achari R, Wesnes KA, Tracy KA (2016): Single dose interaction study of the arginine vasopressin type 1B receptor antagonist ABT-436 and alcohol in moderate alcohol drinkers. *Alcohol Clin Exp Res* **40**:838-845.
3. Liu W, Katz DA, Locke C, Daszkowski D, Wang Y, Rieser MJ, *et al*. (2013): Clinical safety, pharmacokinetics and pharmacodynamics of the 11β-hydroxysteroid dehydrogenase type I inhibitor ABT-384 in healthy volunteers and elderly adults. *Clin Pharmacol Drug Dev* **2**:133-151.
4. Katz DA, Liu W, Locke C, Dutta S, Tracy KA (2015): Clinical safety and hypothalamic-pituitary-adrenal axis effects of the arginine vasopressin type 1B receptor antagonist ABT-436 in healthy adults. *Psychopharmacol* **233**: 71-81.

**Table S1. Baseline characteristics.**

|  | | Mean ± SD | Range |
| --- | --- | --- | --- |
| Age (years) | | 35.5 ± 9.95 | 19-56 |
| Weight (kg) | | 81.8 ± 18.6 | 50-136 |
| Height (cm) | | 174 ± 11.6 | 137-199 |
| Sex (N) | | 33 Male,18 Female | |
| Race (N) | | 32 White,17 Black,1 Asian,1 Other | |
|  | | Placebo | ABT-436 |
| N | | 20 | 31 |
| Current episode duration (months) | | 27.6 ± 50 | 14.9 ± 25 |
| Prior anti-depressant medications during the current episode (N) | Zero | 16 | 30 |
|  | One | 4 | 1 |
| M.I.N.I. primary diagnosis (N) | Recurrent | 13 | 16 |
|  | Single Episode | 7 | 15 |
| Baseline HAM-D-17 (mean) |  | 17.90 | 17.39 |
| Baseline CGI-S (N) | Mildly Ill | 7 | 12 |
|  | Moderately Ill | 13 | 19 |

M.I.N.I. = Mini International Neuropsychiatric Inventory, Version 6.0. HAM-D-17 = Hamilton Depression Rating Scale, 17-item. CGI-S = Clinical Global Impression of Severity. PSS-14 = Perceived Stress Scale, 14 item.

**Table S2. Concomitant medications used by study subjects.**

| **Medication Name** | **Placebo (N=20)** | **ABT-436 (N=31)** | **Total (N=51)** |
| --- | --- | --- | --- |
| **Prior Medications** | | | |
| Cilest | 0 | 2 | 2 |
| Clearasil | 0 | 1 | 1 |
| Ibuprofen | 0 | 2 | 2 |
| Multivitamin | 1 | 0 | 1 |
| Naproxen | 0 | 1 | 1 |
| Paracetamol | 0 | 2 | 2 |
| Zestoretic | 1 | 0 | 1 |
| **Concomitant Medications** | | | |
| Bismuth | 0 | 4 | 4 |
| Cilest | 0 | 2 | 2 |
| Clearasil | 0 | 1 | 1 |
| Ibuprofen | 1 | 3 | 4 |
| Multivitamin | 1 | 0 | 1 |
| Naproxen | 0 | 1 | 1 |
| Paracetamol | 1 | 7 | 8 |
| Zestoretic | 1 | 0 | 1 |

**Table S3. Treatment-emergent adverse events observed in only one subject.**

|  | Placebo | ABT-436 |
| --- | --- | --- |
| N | 20 | 31 |
| Abdominal distension | 0 | 1 (3%) |
| Abdominal pain upper | 0 | 1 (3%) |
| Contusion | 0 | 1 (3%) |
| Dermal cyst | 0 | 1 (3%) |
| Dysgeusia | 0 | 1 (3%) |
| Energy increased | 0 | 1 (3%) |
| Flank pain | 0 | 1 (3%) |
| Influenza-like illness | 0 | 1 (3%) |
| Joint sprain | 0 | 1 (3%) |
| Metrorrhagia | 0 | 1 (3%) |
| Muscle spasms | 0 | 1 (3%) |
| Oropharyngeal pain | 0 | 1 (3%) |
| Orthostatic hypotension | 0 | 1 (3%) |
| Palpitations | 0 | 1 (3%) |
| Panic attack | 0 | 1 (3%) |
| Paraesthesia | 0 | 1 (3%) |
| Tachycardia | 0 | 1 (3%) |
| Vomiting | 0 | 1 (3%) |
| Back pain | 1 (5%) | 0 |
| Dysacusis | 1 (5%) | 0 |
| Dysuria | 1 (5%) | 0 |
| Hot flush | 1 (5%) | 0 |
| Lymphadenopathy | 1 (5%) | 0 |
| Nervousness | 1 (5%) | 0 |
| Neuralgia | 1 (5%) | 0 |
| Toothache | 1 (5%) | 0 |

**Table S4. Central values for saliva measures of hypothalamic-pituitary-adrenal axis activity by regimen.**

|  | Cortisol (ng/mL) | | | Cortisone (ng/mL) | | |
| --- | --- | --- | --- | --- | --- | --- |
|  | Placebo | ABT-436 | P value | Placebo | ABT-436 | P value |
| N | 20 | 31 |  | 20 | 31 |  |
| Waking | 2.08 | 1.26 | 0.004 | 7.71 | 5.40 | 0.001 |
| +30 minutes | 2.36 | 1.93 | 0.238 | 8.70 | 7.61 | 0.217 |
| +60 minutes | 2.00 | 1.56 | 0.157 | 8.85 | 7.54 | 0.140 |
| Average | 2.14 | 1.56 | 0.009 | 8.40 | 6.77 | 0.010 |
| CAR | 2.47 | 1.98 | 0.051 | 9.34 | 7.55 | 0.007 |
| Hour 6 | 0.45 | 0.43 | 0.797 | 4.13 | 3.10 | 0.017 |
| Hour 14 | 0.15 | 0.18 | 0.303 | 1.59 | 1.60 | 0.939 |
| Diurnal | 3.13 | 2.42 | 0.077 | 9.01 | 7.58 | 0.100 |

Average = average cortisol (or cortisone) during the awakening response. CAR = area under the saliva-concentration time curve during the awakening response. Diurnal = difference between maximum and minimum measured levels in an individual.

**Figure S1. Schematic of procedures on Days 6 and 7. The light bulb indicates lights-on time**. The capsule indicates dosing time for 800 mg ABT-436 or matching placebo. Green arrows indicate saliva sample times. Red arrows indicate blood sample times for basal HPA axis assessment. ACTH, cortisol and CBG samples were collected at each time. The middle weight arrows indicate that AVP and copeptin samples were also collected. The heaviest arrows indicate that AVP, copeptin, testosterone, estradiol, dihydroepiandrosterone sulfate and androstenedione samples were also collected. Black arrows indicate blood sample times for dynamic HPA axis assessment. ACTH and cortisol samples were collected at each time. The thicker arrows indicate that cytokine samples were also collected. The yellow lines indicate the time interval for 24-hour urine collection. The blue arrow indicates the time of CRH infusion. The blue line indicates the time interval of IV placement for the CRH challenge. Depression scales (HAM-D-17 and MASQ) were administered after dosing and before lunch, which was served 4 hours after dosing. All Day 6 procedures, except for dosing, were conducted on Day -2. In addition, depression scales were administered on Day -2 at the same time as Day 7. All Day 7 procedures, except for dosing and depression scales, were administered on Day -1.
